# Clinical Use of the Low-Molecular-Weight Heparins in Cancer Patients: Focus on the Improved Patient Outcomes

**DOI:** 10.1155/2011/530183

**Published:** 2011-04-12

**Authors:** Bo H. Chao, Lisa Lepeak, Ticiana Leal, H. Ian Robins

**Affiliations:** ^1^Department of Medicine, University of Wisconsin School of Medicine and Public Health, 600 Highland Avenue, Madison WI 53792, USA; ^2^University of Wisconsin Paul Carbone Comprehensive Cancer Center, 600 Highland Avenue, Madison, WI 53792, USA; ^3^Departments of Medicine, Human Oncology, and Neurology, University of Wisconsin School of Medicine and Public Health, 600 Highland Avenue, Madison, WI 53792, USA

## Abstract

Patients with malignant neoplastic diseases represent a high-risk population relative to thromboembolic disease. With the advent of improved and accessible diagnostic technology, for example, ultrasound and/or spiral CT scans, timely diagnosis of venous thromboembolic events (VTE) is readily accomplished. The introduction of low-molecular-weight heparin (LMWH) approximately two decades ago (in contrast to unfractionated heparin and vitamin K antagonists) has provided a class of agents with a favorable therapeutic index. In the review to follow, the literature regarding the use of LMWH in oncologic patient populations is summarized. Topics addressed include prophylaxis, and treatment as well as consideration of the potential anti-neoplastic properties of this class of drugs.

## 1. Introduction

It is well recognized that cancer patients are at risk for venous thromboembolic events (VTEs) [1] with an estimated annual incidence of VTE in the cancer population five times higher than the general population [2]. This is in part due to chronic hemostatic activation and changes, including increased platelet adhesion and disseminated intravascular coagulation, which are commonly observed in cancer patients [3–10]. Although it is difficult to compare rates across studies, several generalizations regarding VTE can be made. An estimated 15% of all patients with cancer will develop VTE during their clinical course [11]. The risk of developing VTE is significantly higher even early in diagnosis with a reported >50-fold higher incidence in the first 3 months following a diagnosis of cancer in one case controlled study [12]. Patients admitted to an inpatient oncology service are reported to have rates of VTE up to 8% [13, 14]; those who have a remote diagnosis of cancer are at a lower risk, as would be expected [13, 15, 16]. Additionally, other factors may represent more significant risk variables in cancer patients such as certain types of chemotherapy [17, 18]. 

Chemotherapy is associated with a 2- to 6-fold increased risk of VTE referenced to the general population [17–19]. For instance, in a study of 205 patients with stage II breast cancer randomized to 36 weeks of chemotherapy (cyclophosphamide, methotrexate, fluorouracil, vincristine, and prednisone) or 12 weeks of chemohormonal therapy (cyclophosphamide, methotrexate, fluorouracil, vincristine, prednisone, doxorubicin, and tamoxifen), there was a 6.8% incidence of deep venous thrombosis (DVT). Of note, all of the events occurred during the chemotherapy treatment period, suggesting that chemotherapy for breast cancer contributes to thrombosis [20]. A prospective observational study by Khorana et al. in 2007 showed VTE as one of the leading causes of noncancer death among 4,466 patients receiving outpatient chemotherapy, with 9.2% of deaths secondary to VTE and one death related to pulmonary embolism in a patient without known metastatic disease [19]. This is a trend that was again demonstrated in a prospective study of 4,000 patients which showed VTE was an independent risk factor for mortality during the first months of chemotherapy even after adjusting for several risk factors. Cancer diagnosed at the same time or within 1 year after an episode of VTE was associated with worse prognosis and relatively low rate of survival at one year (38 percent versus 47 percent in the control group) [21].

Although specific incidence rates may vary based on the clinical setting, recent reviews have highlighted the significance of histological diagnosis [18, 22]. Patients with cancers of the pancreas [23] and brain [24], in some series, can have rates exceeding 25%; other cancers associated with the highest rates of VTE exceeding 5% include cancers of the gastrointestinal tract, ovary, uterus, kidney, and lung [18]. More recent studies have identified VTE in association with hematologic malignancies as being comparable to solid tumor patients [22]. It is interesting to note oncogenes responsible for neoplastic transformation in leukemia also may be involved in clotting activation [22]. As is the case with solid tumors, therapy for hematologic malignancies can place patients at increased risk. For example, thalidomide [25, 26] and lenalidomide [27, 28] have been associated with high rates of VTE exceeding 25%. In addition to malignancy as a risk factor, other interventions in cancer patients, such as surgery and the use of venous access devices, may further increase the risk for thromboembolic complications [29].

In summary, as VTE contributes to morbidity and is one of the leading causes of noncancer death among patients with cancer [19, 21, 30], the timely diagnosis and effective treatment of VTE is of paramount importance. In the following text we will discuss the existing data for the treatment and prophylaxis of VTE in cancer patients. The review will focus on the use of low-molecular-weight heparins (LMWHs), as the published literature and experience center on this class of anticoagulants in relation to both safety and efficacy. Encompassed in this discussion will be a review of the data suggesting the potential for antineoplastic effects with the application of LMWH, and their influence on patient outcomes. Relative to this, there are *in vitro* and animal studies showing anticoagulants, in particular the LMWHs, have an antineoplastic effect through mechanisms such as interference with tumor cell adhesion, invasion, metastasis formation, and angiogenesis [31–42].

## 2. Low-Molecular-Weight Heparin as Treatment for Venous Thromboembolism

### 2.1. Pharmacology and Pharmacokinetics of Low-Molecular-Weight Heparin

There are various FDA approved LMWHs commercially available which are produced by chemical or enzymatic depolymerization of unfractionated heparin (UFH). The fragmented LMWHs are approximately 1/3 the size of UFH and range from 4,000–6,500 Daltons with a chain length of 12–22 polysaccharides. LMWH's anticoagulant activity is similar to UFH; however, a shortened polysaccharide chain allows for more specific binding to inhibit factor Xa. Unfractionated heparin binds to antithrombin, initiating inhibition of several serine proteases in the coagulation cascade, including factor IIa and Factor Xa. The polysaccharide chain is truncated in LMWHs while retaining the high affinity pentasaccharide binding sites. This results in inhibition of factor Xa, but the shortened polysaccharide chain is relatively ineffective at binding, resulting in loss of inhibition of factor IIa [43]. In terms of anticoagulation, this results in a reduced antifactor IIa activity relative to factor Xa and improved pharmacokinetics. 

Unfractionated heparin is generally given intravenously (if used therapeutically) and requires close monitoring of activated partial thromboplastin time (APTT) with frequent dose adjustments. In contrast, LMWHs are injected subcutaneously once or twice daily and do not typically require routine monitoring. Special considerations for monitoring should be given for patients with renal failure and obesity. LMWHs are cleared by the kidneys, and dosing is weight based. The biological half-life is prolonged in patients with renal failure. Dosing in renal failure and weight adjusted dosing were not addressed in original clinical trials, and monitoring of anti-Xa levels is recommended [44, 45]. Monitoring in pregnancy may also be considered given the relative increase in renal clearance [46]. Anti-Xa levels can be monitored in serum levels 4 hours following subcutaneous administration. Various labs are available for monitoring anti-Xa levels, and the target therapeutic range is 0.6–1.0 IU/mL [47, 48].

### 2.2. Safety and Efficacy

Many consider LMWH as the agent of choice for the initial and long-term treatment of VTE in patients with neoplastic disease, based on randomized clinical studies [19, 49–52]. In 2003, Lee et al. conducted a randomized study of cancer patients with VTE: 336 patients received dalteparin at 200 IU/kg daily for 5 to 7 days followed by a vitamin K antagonist with target international normalized (INR) ratio of 2-3 for 6 months, whereas the other 336 patients received dalteparin at 200 IU/kg daily for 1 month followed by 150 IU/kg daily for 5 months [53]. (It should be noted that the concept of a 25% dose reduction after initial therapy was an arbitrary choice, and not evaluated in comparison to other possibilities.) In this study, the cumulative risk of symptomatic and recurrent VTE during 6 months of anticoagulation was 17% for patients treated with a vitamin K antagonist, compared with 9% for patients treated with dalteparin, a LMWH [53]. The relative risk reduction of 52% was statistically significant (hazard ratio = 0.48; *P* = .002). One episode of recurrent VTE was prevented for every 13 patients treated with dalteparin. In terms of side effects, no difference in major or minor bleeding was detected between the groups, with 6% of dalteparin-treated patients and 4% of control patients having major bleeding episodes [53].

In another study with a similar design, Meyer and colleagues randomized 138 patients to receive either enoxaparin at 1.5 mg/kg daily for 4 days followed by warfarin with target INR of 2-3 for 3 months, or enoxaparin at 1.5 mg/kg daily for 3 months [50]. There was a consistent but not statistically significant benefit with enoxaparin over warfarin, in terms of decreased VTE recurrence or major bleeding [50]. Of note, the experience with LMWH has shown good patient adherence with self-injection [49–51, 53]. Further, it should be noted in comparison to unfractionated heparin, LMWH reduces the risk of recurrent thrombosis by 32%, major bleeding by 43%, and death by 24% [54]. Thus, in considering the aforementioned observations taken collectively, LMWH can be considered a preferred agent for treatment of cancer-associated VTE because of its efficacy, safety, and convenience.

One special case for VTE treatment with LMWH, which deserves special consideration, relates to patients with high grade gliomas, for example, glioblastoma multiforme (GBM). VTE occurs in 20–30% of patients with malignant glioma per year of survival [55, 56]. These patients are also at high risk for bleeding with and without anticoagulation. In one study discussed in detail below (Section 3, Glioma), the incidence of bleeding was 5.1% [57]. In a review on the subject, Chao and Robins suggested treating VTE in patients with GBM with full dose LMWH until resolution of symptoms from their thrombosis (usually within 2 weeks of onset of VTE), followed by 50% dose reduction in the following month, and followed by another 25% dose reduction after 2 to 3 months [58]. They further recommended dividing the total daily dose during the acute phase of therapy into twice-a-day or split dosing to reduce bleeding risk. Parenthetically, one study demonstrated split dosing of dalteparin was more efficacious [59]. Two other studies, one with enoxaparin [60], and one with dalteparin [61], suggested a trend, which was not statistically significant, for better efficacy with split dosing. These authors estimated the incidence of recurrent VTE at less than 5%. Should any patient develop recurrent VTE during dose reduction of LMWH, it was suggested to check the anti-Xa factor levels (as appropriate to the type of LMWH used) after an injection and adjust the dose accordingly [58, 61, 62]. The aforementioned treatment algorithm (which had been extended to other tumor types, e.g., breast cancer patients) was based on experience. The use of a lower dose of LMWH after acute treatment was felt consistent with the efficacy of LMWH in the prophylaxis setting (see also Section 3) and had evolved as a conceptual extrapolation. It has been the practice of many neuro-oncologists to use LMWH indefinitely in high grade glioma patients. This is consistent with the experience in a Eastern Cooperative Oncology Group (ECOG)/Radiation Therapy Oncology Group (RTOG) trial, described in detail in Section 3 below [63].

### 2.3. Other Adverse Side Effects

It is worth noting that patients receiving LMWH for extended periods are at increased risk for osteoporosis, and may have other risk factors for bone demineralization, such as the concurrent use of steroids, hence, monitoring these patients with bone mineral density studies, and considering interventions (e.g., bisphosphonates) as appropriate.

### 2.4. Alternatives to Low-Molecular-Weight Heparin and Warfarin in Treatment of VTE

Fondaparinux, a synthetic selective inhibitor of activated factor X (with no activity against thrombin), is approved for the initial therapy of VTE [64]. In terms of cost and administration convenience, it is comparable to LMWH. Fondaparinux, however, has not been studied specifically in cancer populations. As a result, its use in cancer patients has generally been limited in patients who have a history of heparin-induced thrombocytopenia or allergies to heparin [65].

The use of vena cava filters, as part of treatment of VTE in cancer patients, is not well defined, as previously reviewed [65]. As is obvious, filters do not change the underlying hypercoagulable state in cancer patients. The use of filters in patients receiving anticoagulation has been observed to reduce the incidence of symptomatic pulmonary embolism, but also resulted in more recurrent DVTs and no difference in overall survival [66]. Thus, many clinicians believe filters should be reserved for situations in which anticoagulant therapy is contraindicated, for example, severe thrombocytopenia, active bleeding, or in anticipation of a significant neurosurgical intervention.

## 3. Low-Molecular-Weight Heparin as Prophylaxis for Venous Thromboembolism

Cancer patients are at high risk of VTE, especially in the setting of immobility, hospitalization, or surgery [13, 14, 16, 67]. However, randomized clinical trials of VTE prophylaxis during hospitalization have been conducted in general medical and surgical patients only. There has not been a randomized trial in VTE prophylaxis targeting a hospitalized medical (as opposed to surgical) cancer patient population. There have been three large, randomized, double blind, placebo-controlled trials demonstrating the benefits of anticoagulant prophylaxis, with LMWH or fondaparinux, in general medical patients [68–70]. More than 1,000 hospitalized patients with either congestive heart failure, acute respiratory failure without requiring ventilator support, acute infection, or acute rheumatic disorder in association with an additional risk factor including cancer, were randomly assigned to receive enoxaparin 40 mg, enoxaparin 20 mg, or placebo subcutaneously once daily for 6 to 14 days; in this study, 12.4% of patients had cancer [70]. The incidence of VTE was significantly lower in the group that received enoxaparin 40 mg (5.5%) than in the group that received placebo (14.9%; RR = 0.37; *P* < .001). There was no significant difference between the groups that received 20 mg of enoxaparin and placebo, respectively [70]. Similarly, the incidence of VTE in patients hospitalized with an acute medical condition was reduced from 4.96% in the group that received placebo to 2.77% in the group that received dalteparin 5,000 units subcutaneously once daily, representing a relative risk reduction of 45% (95% CI, 38% to 80%; *P* = .0015) [69]. A subgroup analysis from the first study showed that cancer patients had an elevated risk of VTE and that prophylaxis with 40 mg of enoxaparin daily in cancer patients led to a relative risk reduction of 0.5 (95% CI, 0.14 to 1.72), which was similar to the benefits observed in the whole group, despite being underpowered and not reaching statistical significance [71]. It can be inferred from these studies that VTE prophylaxis with LMWH in hospitalized cancer patients would be beneficial.

For any given site of operation, patients with malignancy undergoing surgery have a higher reported frequency of VTE in the first 90 days after operation [72]. In a multicenter, randomized, and double-blind study, 332 patients undergoing open curative surgery for abdominal or pelvic cancer received one of two durations of postoperative prophylaxis for VTE with enoxaparin 40 mg once daily, either for 28 days postoperatively, or for 6 to 10 days after operation until hospital discharge followed by placebo injections for a further 21 days [73]. The frequency of VTE decreased from 12% in the placebo group to 4.8% in the treatment arm (*P* = .02), based on screening venography at the end of the 28-day period [73]. The benefits of extended VTE prophylaxis were maintained for 3 months after operation without any evidence of a rebound thrombosis [73].

The role of VTE prevention in ambulatory patients with cancer is controversial. In a randomized, placebo-controlled European trial, 353 patients with metastatic breast cancer received either certoparin at 3,000 anti-Factor Xa units daily or placebo for 6 months; the rate of VTE in each treatment group was 4% [74]. In a similarly designed trial, the rate of VTE in 547 patients with stage III or stage IV nonsmall-cell lung cancer was 4.5% in the LMWH arm compared with 8.3% in patients receiving placebo (*P* = .07) [74]. In a subgroup analysis involving patients with stage IV lung cancer, LMWH led to a statistically significant reduction in VTE (10.1% versus 3.5%; *P* = .03). Certoparin is not currently available in the United States. 

The largest ambulatory study to date on VTE prophylaxis was concluded by Agnelli and colleagues [52]. This study addressed the clinical benefit of the LMWH, that is, nadroparin, for the prophylaxis of thromboembolic events in ambulatory patients receiving chemotherapy for metastatic or locally advanced solid cancer. The primary study endpoint was the incidence of symptomatic venous or arterial thromboembolic events. Patients (*n* = 1150) were randomized (2 : 1) between nadroparin and placebo group. Results demonstrated 15 (2.0%) of 769 patients treated with nadroparin and 15 (3.9%) of 381 patients treated with placebo had a thromboembolic event (single-sided *P* = .02). Five (0.7%) of 769 patients in the nadroparin group, and no patients in the placebo group, had a major bleeding event (two-sided *P* = .18). The incidences of minor bleeding were 7.4% (57 of 769) with nadroparin and 7.9% (30 of 381) with placebo. There were 121 (15.7%) serious adverse events in the nadroparin group and 67 (17.6%) serious adverse events in the placebo group.

The CONKO 004 trial addressed chemotherapy with or without LMWH, that is, enoxaparin (1 mg/kg) in patients (*n* = 312) with advanced pancreatic cancer [75]. The primary endpoint was the reduction of symptomatic VTE within the first 12 weeks of treatment. Secondary endpoints were toxicity, time to progression, and overall survival. There were 22 VTE in 152 patients in the observation group and 8 in 160 in the LMWH group. Per-protocol (PP) and intent to treat (ITT) analyses demonstrated significant risk reductions from 14.5% to 5.0% (65% RRR) and 14.5% to 3.8% (74% RRR) for LMWH, respectively. Similarly, the UK FRAGEM study reported a 62% risk reduction in VTE using the CLOT therapeutic regimen of dalteparin (31% versus 12%; *P* = .02) [76]. Major bleeding events were 9.9% for the observation group and 6.3% for LMWH (ITT). In each group there was one tumor-related fatal hemorrhage. Preliminary data demonstrated no difference in time to progression, or survival for observation versus LMWH, respectively, (19 versus 22 weeks) and (29 versus 31 weeks) [75].

### 3.1. Special Considerations for Low-Molecular-Weight Heparin Prophylaxis

#### 3.1.1. Neurosurgery

Prophylaxis in neurosurgical patients has been studied as a unique patient cohort. Two multi-center, randomized, double-blind trials studied the use of LMWH (nadroparin 7500 units subcutaneously once daily, or enoxaparin 40 mg subcutaneously once daily, resp.) versus placebo in conjunction with the use of compression stockings in the prevention of VTE after elective neurosurgery [77, 78]. The majority of the accrued patients had brain tumors, up to 30% of whom were diagnosed with glioma [78]. LMWH or placebo was given within 24 hours after surgery and continued for up to 10 days or until hospital discharge. Both studies showed that LMWH combined with compression stockings was more effective than compression stockings alone for the prevention of VTE after elective neurosurgery, without inducing any significant increase of major bleeding. In the nadroparin study [77] there was a trend for a relative risk reduction in DVT of 28% with a 1.5% increase in nonfatal bleeding. The enoxaparin study [78] demonstrated statistically significant relative risk of 0.52 and no difference in major bleeding risk. However, it should be noted that LMWH in these studies was given within 24 hours after surgery. Another randomized study conducted at the University of Michigan, where LMWH (enoxaparin 30 mg subcutaneously every 12 hours) was initiated before the induction of anesthesia and was continued throughout the hospital stay, showed that enoxaparin therapy initiated at the time of anesthesia induction significantly increased postoperative intracranial hemorrhage [79]. This study was terminated early because of the increased incidence of adverse events in the enoxaparin treatment group. In conclusion, LMWH given within 24 hours after neurosurgery, but not prior to neurosurgery, in combination with compression stockings, can be considered for the prevention of VTE in glioma patients when continued for up to 10 days.

#### 3.1.2. Glioma

As alluded to above, glioma patients are highly predisposed to thromboembolic phenomena with an incidence approaching *∼*30% [55, 56]. In a phase II study, Robins and colleagues studied patients with newly diagnosed GBM: this patient group received dalteparin at 5,000 units daily during and after radiation [63]. The primary endpoint of the study was survival; the secondary end point was the incidence of VTE. This clinical trail closed prematurely (*n* = 42), as standard of care for GBM changed with the introduction of temozolomide [80]. As the study was originally conceived it was estimated that *∼*30% of the enrolled patients would develop VTE. Based on statistical estimates, a study cohort of 72 patients was planned, resulting in 81% power estimate to detect a decrease from 30% to 15% in VTE. When the study was concluded there were no observed VTE in patients actively receiving dalteparin. Time on dalteparin ranged from 1.2 months to 25.4 months, with a median time of 6.3 months. There were no reports of grade 3/4 bleeding or thrombocytopenia related to dalteparin before or aftert progression. There were local site reactions to dalteparin (*∼*71% of patients); 2 patients experienced grade 3 toxicities ascribed to radiation. Two patients developed DVT after stopping dalteparin, at a time when they had developed progressive GBM. 

 A seemingly contradictory result, reported by Perry and colleagues, relates to a phase III Canadian study in which 186 malignant glioma patients were randomized to dalteparin at 5,000 units daily versus placebo. Unfortunately, this trial also closed prematurely, due to lack of drug availability. It was planned to enroll 512 patients. In this study the primary endpoint was the incidence of VTE. There was a trend for a decrease in VTE; however, it was not statistically significant (HR = 0.51, 95% CI: 0.19–1.4, *P* = .29). There was also a trend for increased bleeding in the LMWH arm, that is, 5.1% (*n* = 5) versus 1.2% (*n* = 1) for the placebo. The clinical significance of this finding is uncertain, as intratumoral hemorrhage is often part of the natural history of this disease [57]. Noteworthy differences in these studies may relate to differences in patient populations studied. The RTOG study was restricted to GBM patients (as opposed to all malignant glioma); there was also a requirement for an unusually good performance status, that is, 95% ECOG performance status of 0 or 1 [57]. 

The results of these two studies, taken collectively, leave the role of thromboembolic prophylaxis with an anticoagulant as undefined for this group of patients with central nervous system disease. As a result, further investigation is required to reach definitive conclusions regarding the application of VTE prophylaxis beyond the postoperative period after hospital.

## 4. Coagulation as a Host Factor in Cancer

The association of disseminated intravascular coagulopathy (DIC) and cancer is well documented in various malignancies, including melanoma, breast, colon, gall bladder, ovary, prostate, and stomach cancers [81]. This association can be explained by general factors related to the host's response to cancer and, more specifically, to factors related to procoagulant activities expressed by the cancer cells [82]. Thrombosis occurs in 5–15% of patients with cancer [83], comparing to 0.1% in the general population [84].

Indeed, the occurrence of venous thrombosis in an otherwise healthy person may be the first sign of an occult malignancy [85–87], as malignancy is often associated with thromboembolic disease [81]. Investigations have shown that fibrin is abundant in the stroma of many tumors [88–90]. The long-observed association of clotting with malignancy prompted scientists over 30 years ago to test the hypothesis that coagulation played an adjunctive role in cancer progression. As reviewed by Zacharski and colleagues, a number of experimental animal studies (and limited number of small-scale clinical trials in cancer patients) suggested that anticoagulants, including heparin and warfarin, might successfully inhibit tumor spread [91]. While a role for LMWHs (and perhaps heparin in general) in cancer treatment can be logically argued based on theories of fibrin and tumor implantation, heparin may have important antitumor effects which are independent of their anticoagulant properties. Folkman and colleagues, for example, found that heparin fragments lacking evident anticoagulant activity could still effectively inhibit angiogenesis in presence of cortisone [92]. Other investigators have reported that, individually, high-molecular-weight fractions of heparin stimulate, while low-molecular-weight fractions inhibit, angiogenesis and that this effect was independent of anticoagulation [93]. Furthermore, Bitan and colleagues have shown that the ability of heparin and heparin fragments to inhibit metastasis of melanomas, by inhibiting heparinase-dependent degradation of the extracellular matrix, was the same in both anticoagulant and non-anticoagulant fractions [94]. As cancers are most typically manifested by uncontrolled cellular proliferation, it may also be relevant that both anticoagulant and non-anticoagulant fractions of heparin and heparin fragments can inhibit the proliferation of vascular smooth muscle cells [95]. Collen and colleagues have demonstrated the inhibitory effect of heparins on the proliferation of human microvascular endothelial cells, which provides a novel mechanism by which LMWH may affect tumor progression, namely, reduced ingrowth of microvascular structures in a fibrinous stromal matrix by rendering it less permissive for invasion [96].

## 5. Anticoagulation and Cancer Survival

It is of historical interest to note that in 1976 the Veterans Administration (VA) Cooperative Study Program initiated a prospective randomized clinical trial of warfarin in cancer patients to explicitly test the hypothesis that coagulation was involved in cancer progression [91]. In providing a rationale for their trial, the investigators proposed that clotting may (1) facilitate attachment of metastatic tumor cells to the endothelium; (2) provide growth factors for the tumor; (3) provide a structural lattice for cell proliferation; (4) provide tumor cells protection from host immune defenses [91]. As reported in 1984, the group found no differences in survival between warfarin-treated and control groups for several cancers. However, warfarin therapy was associated with a significant prolongation in the time to first evidence of disease progression (*P* = .016) and a significant improvement in survival (*P* = .018) for patients with small cell carcinoma of the lung, including the subgroup of patients with disseminated disease at the time of randomization (*P* = .013) [97].

Interest in the potential relationship between neoplastic diseases and coagulation followed the publication of reports suggesting improved prognosis in cancer patients with thrombosis receiving low-molecular weight-heparin (LMWH) [98]. A meta-analysis of a number of randomized clinical trials suggested the superiority of LMWH over standard heparin in subgroups of patients with cancer [98]. Two early studies from this literature also suggest an unexpected and unexplained benefit of LMWH as compared to unfractionated heparin treatment: an apparent reduction in cancer-related mortality in the first three months following initiation of treatment [86, 99]. A review of published clinical trials involving LMWH as treatment and prophylaxis for venous thrombosis analyzed a few other studies with data on cancer-related mortality [100]. Although many LMWH studies have reported patients' baseline characteristics, which sometimes included cancer, the follow-up periods were not of significant duration to allow a meaningful assessment of treatment effect on mortality. A meta-analysis of LMWH versus unfractionated heparin (UFH) studies found the 90-day mortality in patients with cancer in four studies to be 14% in the LMWH group, and 28% in the heparin group (*P* = .01) [86, 99–102].

It is of interest to note that in the aforementioned CLOT study (in Section 2 above), which was a randomized trial of dalteparin for 6 months compared to dalteparin for a brief initial period followed by a vitamin K antagonist, survival was assessed as a secondary outcome; the primary outcome was rate of recurrent thrombosis [53]. Survival was not found to be improved in the dalteparin arm compared with the control arm. However, a post hoc subgroup analysis, focusing only on the patients with nonmetastatic disease, reported the 12-month all-cause mortality of 35% in vitamin K antagonist treated patients compared to 20% in the dalteparin group (*P* = .04). Conversely, in patients with metastatic cancer, no survival difference was observed between dalteparin (72%) and oral anticoagulant (69%) study arms (*P* = .46) [103].

LMWH has also been studied as an antineoplastic agent. A randomized controlled study (*n* = 84) found a 4-month improvement in progression free survival (*P* = .01) in patients with small cell lung cancer patients receiving combination chemotherapy with LMWH compared to those who did not receive LMWH [104]. In another randomized, placebo-controlled study (The Famous Trial) evaluated 385 patients with advanced malignancy receiving standard treatment with or without dalteparin. Although there was no statistically significant benefit overall in the dalteparin arm, in a post hoc landmark analysis of 102 patients still alive at 17 months, a significant improvement in survival was observed for patients receiving dalteparin [105]. A similar observation was also made in a small study of GBM patients reviewed in Section 3 [63], where better survival was seen in patients maintained on dalteparin compared to those who stopped dalteparin after first disease progression. In another randomized study (MALT) (*n* = 302) using nadroparin, a significant improvement in overall survival was observed (relative risk, 0.75; 95% CI, 0.59 to 0.96; *P* = .02) [106]. However, a similar but smaller phase 3 study (*n* = 144) using dalteparin was a negative trial [107]. 

The aforementioned laboratory and clinical studies are a sampling of the mounting evidence that implicates components of the clotting cascade and neoplastic tumor progression. An excellent review of the subject has been written by Kuderer and colleagues [34]. We agree with these authors that LMWH and related agents hold promise for improving cancer outcomes.

## 6. Conclusions

VTE is a significant source of morbidity and mortality for patients with cancer. LMWHs are safe and effective in treatment and prophylaxis of VTE. Continued research regarding the antineoplastic activity of LMWH alluded to above may provide valuable insights into cancer pathogenesis and new treatment paradigms. Future controlled clinical trials directed at evaluating antithrombotic agents relative to treatment and prophylaxis are clearly warranted in this high-risk population. Other areas for needed research include markers and defined patients pathways to better define patient cohorts who are most likely to develop VTE.
